# A polyclonal antibody against a recombinantly expressed *Triticum aestivum* RHT-D1A protein

**DOI:** 10.1186/s43141-020-00072-4

**Published:** 2020-09-16

**Authors:** Izat Smekenov, Sanzhar Alybayev, Temurkhan Ayupov, Guliza Rakhmatullaeva, Amangeldy Bissenbaev

**Affiliations:** 1 Department of Molecular Biology and Genetics, Faculty of Biology and Biotechnology, al-Farabi Kazakh National University, Almaty, Kazakhstan 050040; 2 Scientific Research Institute of Biology and Biotechnology Problems, al-Farabi Kazakh National University, Almaty, Kazakhstan 050040

**Keywords:** *Triticum aestivum*, RHT-D1A, DELLA, Norin 10, Saratovskaya 29, Polyclonal antibody

## Abstract

**Background:**

Reduced height-1 dwarfing alleles affect DELLA proteins belonging to a family of putative transcriptional regulators that modulate plant growth and development. The *Arabidopsis thaliana* genome encodes five DELLA proteins, whereas monocot plants, such as rice, barley, and wheat, each have a single DELLA protein. In wheat, wild-type *Rht-B1a* and *Rht-D1a* genes encode DELLA proteins and have many alleles that contain lesions. Among them, *Rht-B1b* and *Rht-D1b* are the most common mutant dwarfing alleles, which have played a key part in the creation of high-yielding wheat varieties. Despite their fundamental roles in plant biology, until now, DELLA proteins in wheat have been mainly researched regarding the phenotypic effect of defective *Rht* mutants on yield-related traits, without studies on the underlying mechanisms. The RHT-1 protein has yet to be detected in wheat tissues, owing to a lack of appropriate molecular tools for characterization of RHT function and protein interactions in signal transduction. This study is focused on the production of a polyclonal antibody to the wheat RHT-D1A protein.

**Results:**

To generate the anti-RHT-D1A antibody, we expressed and purified soluble 6xHis-tagged RHT-D1A. The purified recombinant RHT-D1A was injected into New Zealand white rabbits to generate polyclonal antiserum. The polyclonal anti-RHT-D1A antibody was purified by ammonium sulfate precipitation, followed by affinity chromatography on protein A–agarose beads. The purified polyclonal antibody was demonstrated to be effective in immunoblotting, western blot hybridization, and immunoprecipitation. In wheat seedling extracts, the polyclonal antibody recognized a protein with a molecular mass close to the predicted molecular weight of the endogenous RHT-D1A protein. We also demonstrated that RHT-D1A disappears in response to exogenous and endogenous gibberellic acid.

**Conclusion:**

The purified polyclonal antibody raised against the recombinant RHT-D1A protein is sufficiently specific and sensitive and could be a useful tool for future insights into upstream and downstream components of DELLA-regulatory mechanisms in wheat plants.

## Background

Dwarfing alleles (reduced height, *Rht*) are an important breeding tool for increasing wheat grain yields. *Rht* genes containing lesions are most extensively used in worldwide wheat breeding programs. Among them, mutations *Rht-B1b* and *Rht-D1b* were the major factors in the Green Revolution, and more than 70% of the wheat cultivars all over the world carry at least one of them [1]. *Rht-B1b* and *Rht-D1b* homeoalleles originate from a Japanese variety, Norin 10, and were successfully exploited in wheat breeding programs in the 1950s [2, 3]. Alternative semidwarfing alleles *Rht-B1d* and *Rht-B1e* have found some limited commercial applications, while more strongly dwarfing alleles, such as *Rht-B1c* and *Rht-D1c*, have not so far been exploited because they reduce biomass and the crop yield [4]. Both *Rht-B1b* and *Rht-D1b* involve a point mutation giving rise to a TAG stop codon in the N-terminal coding region [5]. These mutations decrease the plant’s capacity to respond to gibberellic acid (GA), and wild-type plant height is not restored by exogenous application of this hormone. Wild-type *Rht* genes are orthologous to *Arabidopsis* genes *RGA* and *GAI*, which are negative regulators of the GA response [6]. Genes *RGA* and *GAI* encode DELLA proteins, which are transcriptional regulators suppressing the GA signal transduction pathway. The mechanism of DELLA protein–mediated GA signaling in *Arabidopsis* has been elucidated via biochemical, genetic, and structural analyses [7]. It is assumed that SLR1 (Slender Rice-1) in rice and SLN1 (Slender 1) in barley perform a function similar to that of RGA and GAI [8]. In the nucleus, a wild-type *Arabidopsis* DELLA protein binds to GA receptor GID1, GA, and the SCF E3 ubiquitin ligase complex. Such a large complex is recognized by the 26S proteasome and is destroyed. The degradation of DELLA proteins induces GA-responsive plant processes such as seed germination, stem and root elongation, and fertility [7].

DELLA research in wheat has focused on phenotypic effects of *Rht-B1b* and *Rht-D1b* on yield-related traits, without studies on the underlying mechanisms [7]. In wheat, although an interaction between RHT-1 and GID1 has been observed in yeast two-hybrid experiments [9], the RHT-1 protein has yet to be detected in plant tissues, owing to a lack of appropriate molecular tools. To detect the RHT-1 protein in wheat tissues, an antibody specific for this protein is needed. There are currently no commercial high-specificity anti-RHT-1 antibodies available.

In this study, the wheat *Rht-D1a* cDNA gene was synthesized and expressed in *E. coli*, then a polyclonal antibody was generated using the recombinant protein as an antigen, and the suitability of this polyclonal antibody for immunoblotting, western blot hybridization, and immunoprecipitation (IP) was analyzed.

## Methods

### Plant material

Wheat varieties Saratovskaya 29 and Norin 10 were identified and kindly provided by Dr. M.A. Yessimbekova, Department of the Field Crops Gene Pool of Kazakh Research Institute of Agriculture and Plant Growing, Almaty, Kazakhstan (no voucher specimen of this material has been deposited in a publicly available herbarium).

### DNA extraction and PCR analysis

Genomic DNA was isolated from fresh leaf tissues of 4-day-old wheat seedlings using the TRIzol reagent (Invitrogen) following the manufacturer’s instructions. Alleles *Rht-B1b* and *Rht-D1b* were detected as recommended by Ellis et al. [10] by means of a primer combination specific for tall and dwarfing alleles (Table 1). The PCR products were separated on 2% agarose gels and visualized after ethidium bromide staining by standard procedures [11].

**Table 1:** PCR primers related to genes *Rht-B1b* and *Rht-D1b*

Primers	DNA sequences
BF	5′-GGTAGGGAGGCGAGAGGCGAG-3′
DF	5′-CGCGCAATTATTGGCCAGAGATAG-3′
DF2	5′-GGCAAGCAAAAGCTTCGCG-3′
MR1	5′-CATCCCCATGGCCATCTCGAGCTA-3′
WR1	5′-CATCCCCATGGCCATCTCGAGCTG-3′
MR2	5′-CCCCATGGCCATCTCGAGCTGCTA-3′
WR2	5′-GGCCATCTCGAGCTGCAC-3′
Rht_*Eco*RI_F	5′-ATTAAGAATTCTAATGAAGCGGGAGTACCAGGACGCCGGA-3′
Rht*_Hind*III_R	5′-TATTCAAGCTTTCACGGCCCGGCCAGGCGCCAT-3′

### cDNA synthesis

Seeds were surface-sterilized by washing first with 70% ethanol for 2 min, then with 1% sodium hypochlorite for 30 min, and finally with sterile distilled water. The sterilized seeds were next grown at 20 °C on moistened filter paper. Total RNA was extracted from 100 mg of fresh leaf tissues of wheat (*Triticum aestivum*) variety Saratovskaya 29 in liquid nitrogen using the TRIzol reagent (Invitrogen) according to the manufacturer’s instructions. Intactness and high quality of RNA were confirmed by the presence of two intense 28S and 18S ribosomal RNA bands in ethidium bromide–stained agarose gels visualized under UV light. Five micrograms of DNA-free total RNA was converted into single-stranded DNA by means of a mix of oligo-dT_18_ primers and the First Strand cDNA Synthesis Kit (Thermo Scientific). PCR was carried out using 2 μL of a 20-fold dilution of the cDNA, 15 pmol of each primer, and 1 U of Taq polymerase in a 25-μL reaction volume. To generate the cDNA for full-length *Rht-D1a*, the coding sequences were PCR-amplified with primers Rht_*Eco*RI_F and Rht*_Hind*III_R, which were designed based on a previously published mRNA sequence (GenBank accession number HE585643.1; Table 1). The amplified fragments of *Rht-D1a* were cloned into the pBluescript II SK (+) vector at *Eco*RI and *Hind*III restriction sites using the Rapid DNA Ligation Kit (Thermo Scientific). Colonies of transformed *E. coli* DH5α cells carrying plasmids with an insert were screened out by *lacZ* complementation, and the plasmid DNA was isolated with the GeneJET Plasmid Miniprep Kit (Thermo Scientific). The inserts were sequenced in both directions with forward and reverse M13 primers.

### Expression and purification of RHT-D1A proteins

To generate an expression plasmid for 6xHis-tagged RHT-D1A, the full-length coding sequence of *Rht-D1a* was excised with restriction enzymes *Eco*RI and *Hind*III from plasmid pBluescript II SK (+)-*Rht-D1a* and was subcloned into the pET-28c vector at the same sites. The resulting expression plasmid, pET-28c-*Rht-D1a*, yields the respective protein with an N-terminal 6xHis-tag sequence. *E. coli DH*5α served as a cloning host for the propagation of the expression vector. Three expression strains of *E. coli*, BL21(DE3), Rosetta-gami 2(DE3), and ArcticExpress (DE3), were prepared and transformed by the standard protocols [12].

The RHT-D1A protein was purified from a cell extract of IPTG-induced *E. coli* strain ArcticExpress (DE3) (Agilent Technologies) carrying the plasmid with the *Rht-D1a* gene. Briefly, the transformed *E. coli* cells were grown to optical density at 600 nm (OD_600_) of 0.6 at 37 °C, then the temperature was reduced to 13 °C, and the cells were incubated for 30 min. After the desired temperature was reached, 500 μM IPTG was added, and expression was continued for 16 h. Due to strong expression in ArcticExpress (DE3), it was possible to purify RHT-D1A to homogeneity by only single-step affinity purification.

All purification procedures were carried out at 4 °C. For purification of recombinant RHT-D1A (rRHT-D1A) in the native nondenatured state, the bacteria were harvested by centrifugation, and the cell pellets were lysed with a French press at 124.1 MPa in a buffer consisting of 50 mM Tris-HCl (pH 9.0), 100 mM NaCl, 1 mM EDTA (pH 8.0), 5% of glycerol, 1 mM DTT, 10 mM β-mercaptoethanol, and 2% of Triton X-100 and supplemented with the Complete Protease Inhibitor Cocktail (Roche Diagnostics, Switzerland). The EDTA was intended to inhibit proteases, and β-mercaptoethanol and DTT were necessary for the maintenance of a reducing environment. Milder nonionic detergent Triton X-100 is good at solubilizing membrane proteins and for isolation of cytoplasmic proteins. Proteins retain their native conformation in the presence of this detergent. The lysates were cleared by centrifugation at 40,000 × *g* for 60 min at 4 °C, the buffer of the resulting supernatant was adjusted to 500 mM NaCl and 20 mM imidazole, and the sample was loaded onto a HisTrap HP column (GE Healthcare) charged with Ni^2+^. The bound proteins were eluted in a 20–500 mM imidazole gradient. The purified protein samples were stored at − 20 °C in 50% glycerol. The homogeneity of the protein preparations was verified by SDS-PAGE.

### Preparation of the polyclonal anti-RHT-D1A antibody

This antibody was raised against the full-length recombinant 6xHis-tagged RHT-D1A protein (rRHT-D1A). For primary immunization, we subcutaneously injected ~ 1 mg of the purified rRHT-D1A protein mixed with an equal volume of Freund’s complete adjuvant (F5881, Sigma-Aldrich, Canada) at five spots on the dorsal back of a rabbit. Then, reimmunization was done three times with 1 mL of a solution containing 0.5 mg of the purified rRHT-D1A protein in 0.5 mL of elution buffer and 0.5 mL of incomplete Freund’s adjuvant (F5506, Sigma-Aldrich, Canada) with a 14-day interval. One week after the last injection, blood was collected, and ammonium sulfate was added to 3 mL of the obtained rabbit antiserum to achieve 50% saturation. The precipitate was collected by centrifugation, and the pellet was dissolved in purified water and dialyzed against 10 mM potassium phosphate buffer (pH 7.0). The obtained immunoglobulin fraction was applied to a column with protein A–agarose beads equilibrated with the above buffer. After a wash with the same buffer, antibodies were eluted with 100 mM glycine buffer (pH 3.0). The IgG-containing fractions were pooled, and pH was adjusted to 7.0 with 1.0 M Tris base. The resulting suspension was kept at 4 °C.

### Antiserum titer determination by an ELISA

The titers of antisera were determined by an indirect ELISA. Each well of a 96-well ELISA plate (Corning Inc., USA) was coated with 1 μg of rRHT-D1A dissolved in 100 μL of 50 mM carbonate-bicarbonate buffer (pH 9.6) and incubated overnight at 4 °C. After three washes with phosphate-buffered saline (PBS) Tween buffer (PBST; 0.05% of Tween 20 in PBS, pH 7.4), the wells were blocked with 100 μL of 3% BSA in PBST for 1 h at 37 °C and then washed again twice with PBST. After blocking, 100 μL of serially diluted anti-RHT-D1A serum (1:1000 to 1:128000) was added into the antigen-coated wells. The plate was covered with an adhesive plastic and incubated for 2 h at room temperature and then washed four times with PBST. At the next step, a 1:30,000-diluted alkaline phosphatase–conjugated goat anti-rabbit IgG antibody (Sigma, Canada) was added at 100 μL/well and incubated for 1 h at 37 °C. After a wash, 100 μL of a freshly prepared p-Nitrophenyl phosphate substrate solution was added into each well, and the plate was incubated at room temperature in a dark place. Finally, an absorbance was measured at 405 nm (*A*_405_) on a multiskan FC (Thermo Scientific, MA, USA). All samples were tested in triplicate, with each plate containing control wells with positive serum samples and control wells with negative reference serum.

### A dot blot assay

Next, 0.5–1.0 μg of each protein preparation was spotted onto a dry polyvinyl difluoride membrane (Pierce PVDF Transfer Membrane) and dried. The membranes were blocked with 5% nonfat dry milk in 1× Tris-buffered saline containing Tween 20 (TBST; 50 mM Tris-HCl pH 7.6, 150 mM NaCl, 0.005% of Tween 20) for 1 h at room temperature. After that, the membranes were incubated in blocking buffer containing the anti-RHT-D1A (1:10,000 dilution in the blocking solution with 0.1% of Tween 20) or anti-Ape1L polyclonal antibody (1:5000 dilution in the blocking solution with 0.1% of Tween 20) on a rocker overnight at 4 °C. The membranes were then washed thrice in 1× TBST and incubated with a goat anti-rabbit antibody (ab6702, Abcam, Cambridge, UK) at a 1:20,000 dilution on a rocker for 1 h at room temperature. Next, each membrane was washed five times in 10 mL of 1× TBST for 5 min each time. A working substrate solution was prepared by mixing equal volumes of a H_2_O_2_ solution and luminal/enhancer solution. Each membrane was incubated in the working solution for 2 min in darkness, and Kodak X-Omat was exposed to the membrane. The 6xHis-tagged RHT-D1A protein was also detected with the anti-His antibody raised in rabbits (1:1000 dilution; sc-803; Santa Cruz Biotechnology) and an anti-rabbit Ig horseradish peroxidase–conjugated antibody (1:10,000 dilution).

### Plant protein extraction

Wheat grains were sterilized in 2% (v/v) NaOCl for 20 min and washed twice with sterile water, once with 0.01 M HCl, and then thoroughly with sterile distilled water. The grains were allowed to germinate at room temperature on sterile filter paper soaked in water or 10 μM GA in the presence or absence of 100 μM paclobutrazol (PBZ). After 4 days, the seedlings were excised from the seeds and the de-embryonated seeds were ground up in liquid nitrogen and then resuspended in RIPA buffer consisting of 50 mM Tris-HCl (pH 7.6), 2 mM DTT, 1 mM phenylmethylsulfonyl fluoride, 1 mg/mL leupeptin, and 1 mg/mL pepstatin; the cell debris were pelleted, and the protein concentration was determined with the Bradford Protein Assay Kit (Bio-Rad, France).

Total protein samples (25 μg) from each extract were fractionated by SDS-PAGE in a 10% gel and analyzed by western blotting with a 20,000-fold dilution of the anti-RHT-D1A polyclonal antiserum and a 30,000-fold dilution of a peroxidase-conjugated goat anti-rabbit IgG antibody (ab6702, Abcam, Cambridge, UK). The protein extracts were also subjected to detection of α-amylase with a polyclonal antibody against wheat α-amylase, kindly provided by Dr. A. Khakimzhanov (Aitkhozhin Institute of Molecular Biology and Biochemistry, Kazakhstan).

### Zymogram analysis of α-amylase

For this purpose, nondenaturing polyacrylamide gel electrophoresis (native PAGE) was performed according to the method presented by Laemmli [13]. Four-day-old endosperm of wheat seedlings were ground up in liquid nitrogen and then resuspended in 10 mM CaCl_2_. The samples were mixed with 50% saccharose and loaded onto a polyacrylamide gel (4% and 10% polyacrylamide for the stacking and resolving gels, respectively). Electrophoresis was conducted at 100 V and 4 °C. After that, the gel was incubated in 10 mM CaCl_2_ for 30 min at room temperature. Next, the gel was incubated in a 1% (w/v) starch solution at 30 °C and shaken for 60 min. After washing of the gel with distilled water, it was stained with the Lugol solution (1.3% I_2_ and 3% KI). The signals of α-amylase activity appeared as bright bands on a dark background and were photographed.

### Western blotting

Next, 0.5–1.0 μg of purified proteins or 5–10 μg of cell lysates was separated by SDS 10% PAGE. The gel was then electroblotted onto a PVDF membrane in a Bio-Rad Mini-transblot Cell according to the manufacturer’s instructions. After that, each membrane was gently shaken in the blocking solution consisting of 5% milk and 0.1% Tween 20 in 1× TBS (Tris-buffered saline: 50 mM Tris-HCl pH 7.5, 20 mM NaCl) for 1 h at room temperature. After the removal of the blocking solution, the membrane was incubated in 10 mL of a solution of the affinity-purified polyclonal anti-RHT-D1A antibody (1:10,000 dilution in the blocking solution with 0.1% of Tween 20) or the anti-Ape1L polyclonal antibody (1:10,000 dilution in the blocking solution with 0.1% of Tween 20) overnight at 4 °C. The membrane was washed five times in 10 mL of wash buffer (1× TBS with 0.1% of Tween 20), for 5 min each time. After that, the membrane was incubated in 10 mL of a secondary antibody solution (1:20,000 dilution in the blocking solution with 0.1% of Tween 20) for 1 h at room temperature. Then, the membrane was washed five times in 10 mL of wash buffer, for 5 min each time. The working substrate solution was prepared by mixing equal volumes of an H_2_O_2_ solution and luminal/enhancer solution and was used at 0.1 mL per cm^2^ of the blot area. The membrane was incubated in the working solution for 2 min in darkness, and Kodak X-Omat was exposed to the membrane. The 6xHis-RHT-D1A protein was also detected with an anti-His antibody raised in rabbits (1:1000 dilution) and an anti-rabbit Ig horseradish peroxidase–conjugated antibody (1:10,000 dilution).

### Immunoprecipitation

*E. coli* cells expressing the rRHT-D1A protein were grown to OD_600_ of 0.6 at 37 °C, then the temperature was reduced to 13 °C, and the cells were incubated for 30 min. After the desired temperature was reached, 50 μM IPTG was added, and expression was continued for 4 h. The cells were pelleted in lysis buffer (50 mM NaH_2_PO_4_, pH 7.4, 150 mM NaCl, 1 mM EDTA, 5% of glycerol, 0.2% of NP-40, 10 mM MgCl_2_, 1 U of DNase I, 2 mg/mL RNase, 0.1 mg/mL lysozyme, and the protease inhibitor cocktail). The suspension was incubated at 37 °C for 30 min and sonicated thrice for 20 s at 1-min intervals on ice. Cell debris were removed by centrifugation at 10,000×*g* for 5 min. The cell lysates were diluted 1:10 with lysis buffer. For IP, 4 μg of the anti-RHT-D1A polyclonal antibody was added to the cleared cellular lysates containing 1 mg of total protein followed by incubation with rotation at 4 °C for 2 h. After 1-h incubation with 45 μL of a 25% protein G–agarose slurry (Thermo Scientific, MA, USA), immunoprecipitates were washed four times with lysis buffer and analyzed by western blotting.

## Results

### PCR analysis of wheat varieties and the synthesis of the *Rht-D1a* cDNA gene

In wheat, three DELLA genes, *Rht-A1*, *Rht-B1*, and *Rht-D1* (on chromosomes 4A, 4B, and 4D, respectively), are known and share high sequence similarity with one another. *Rht-B1* and *Rht-D1* have many alleles that differ from the wild-type alleles by single-nucleotide substitutions. Among them, *Rht-B1b* and *Rht-D1b* are the most common mutant dwarfing alleles, which have played a key role in the creation of high-yielding wheat varieties. They are now present in more than 70% of current commercial wheat cultivars [1]. Both alleles *Rht-B1b* and *Rht-D1b* involve a point mutation generating a TAG stop codon in the N-terminal coding region [5].

**Fig. 1:**
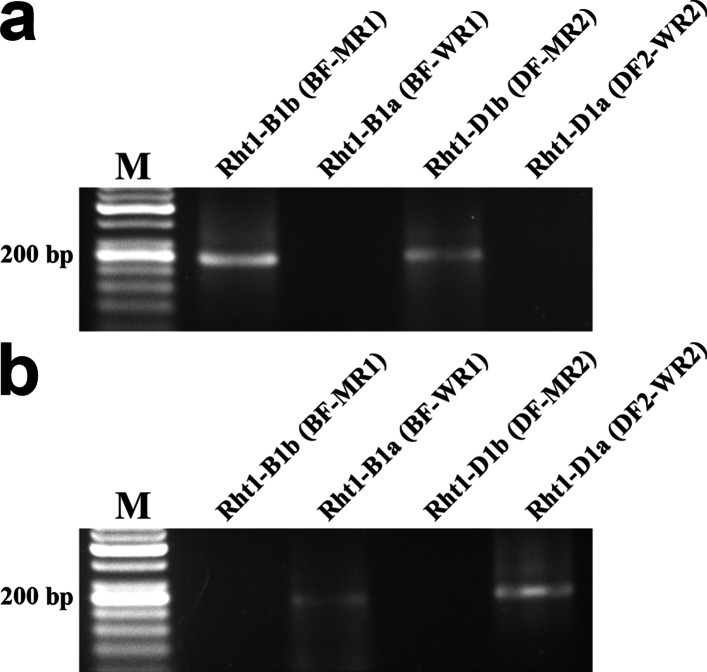
PCR analysis of wheat varieties Norin 10 (**a**) and Saratovskaya 29 (**b**). PCR products were separated on 2% agarose gels after amplification with the following primer sets: BF-MR1, BF-WR1, DF-MR2, and DF2-WR2. The expected product sizes are 237 bp for BF-MR1 and BF-WR1, 254 bp for DF-MR2, and 264 bp for DF2-WR2

This point mutation may cause amino acid misincorporation and/or truncation of the polypeptide, thus affecting correct heterologous expression of the RHT-D1A protein. Furthermore, this point mutation lies within the DELLA domain and thereby may alter the epitopes recognized by the immune response. For these reasons, before synthesizing the cDNA gene of RHT-D1, we decided to test for the presence of these mutant dwarfing alleles in wheat variety Saratovskaya 29. Previously, Ellis et al. designed primers for detecting alleles *Rht-B1b* and *Rht-D1b* [10]. This PCR-based method can detect the point mutations responsible for the two major semidwarfing alleles *Rht-B1b* and *Rht-D1b* in wheat. To test for the presence of these dwarfing alleles, DNA samples from Saratovskaya 29 were analyzed by PCR with primer combinations specific for the tall and dwarfing alleles (Table 1 and Fig. 1). As a positive control, we used double-dwarf wheat variety Norin 10 carrying alleles *Rht-B1b* and *Rht-D1b.* As expected, the double-dwarf wheat variety tested positive with the *Rht-B1b-* and *Rht-D1b-*specific primers (Fig. 1a). By contrast, wheat variety Saratovskaya 29 yielded an amplicon of the expected size (237 bp) with the primer combination specific for tall alleles *Rht-D1a* and *Rht-B1a*; no amplification was detectable with the *Rht-B1b*- and *Rht-D1b*-specific primers (Fig. 1b). Thus, these data indicated that the Saratovskaya 29 wheat variety does not carry mutant alleles *Rht-B1b* and *Rht-D1b*.

A cDNA containing a gene for the putative RHT-D1A protein was obtained as described in the “Methods” section. The cDNA was subcloned at the *Eco*RI and *Hind*III sites into pBluescript II SK (+) and subjected to DNA sequencing. The cDNA consists of 1872 bp and contains a single open reading frame predicted to code for a protein of 623 amino acid residues. The calculated molecular weight of RHT-D1A is 65.3 kDa. Alignment of the RHT-D1A protein sequence revealed 100% similarity with the translated sequences of previously published *Rht-D1a* mRNA (GenBank accession number HE585643.1).

Consequently, in addition to the results of PCR analysis with allele-specific primers, sequencing of synthesized cDNA of RHT-D1A with subsequent alignment of translated sequences confirmed that the chosen wheat variety does not carry mutant alleles *Rht-B1b* and *Rht-D1b*.

### Plasmid construction and optimization of *Rht-D1a* gene expression

The most widely used recombinant systems for obtaining immunogenic antigens are fusion proteins tagged with glutathione S-transferase (GST) [14]. Nonetheless, some animals have antibodies reacting with the GST part of the fusion protein; hence, all obtained antisera have to be checked not only against the recombinant antigen but also against GST alone. The polyhistidine tag is widely and preferably used for recombinant-immunogen production because of its low immunogenicity and rather weak interference with the protein’s function or activity [15]. Therefore, we decided to use a histidine-tagged expression system for rRHT-D1A production. The *Rht-D1a* gene expression vector encoding the 6xHis-tag at the protein’s N terminus was constructed as described in the “Methods” section. The expression plasmid with the cloned *Rht-D1a* gene was designated as pET-28c-*Rht-D1a*. To achieve strong immunological responses against rRHT-D1A, it is desirable to express it as a highly soluble protein. With the aim to improve the recombinant-protein folding and solubility, at first, three *E. coli* expression strains were tested. *E. coli* BL21 (DE3) is the most common prokaryotic strain used for the expression of recombinant proteins [16]. Rosetta-gami 2(DE3) is a strain engineered to enhance the expression of genes containing rare codons. Besides, it can promote disulfide bond formation to stabilize the recombinant protein owing to mutations of genes *trxB* and *gor* [17, 18]. ArcticExpress (DE3) competent cells have been engineered to increase the recovery of a soluble protein because of their adaptation to low-temperature cultivation [19].

**Fig. 2:**
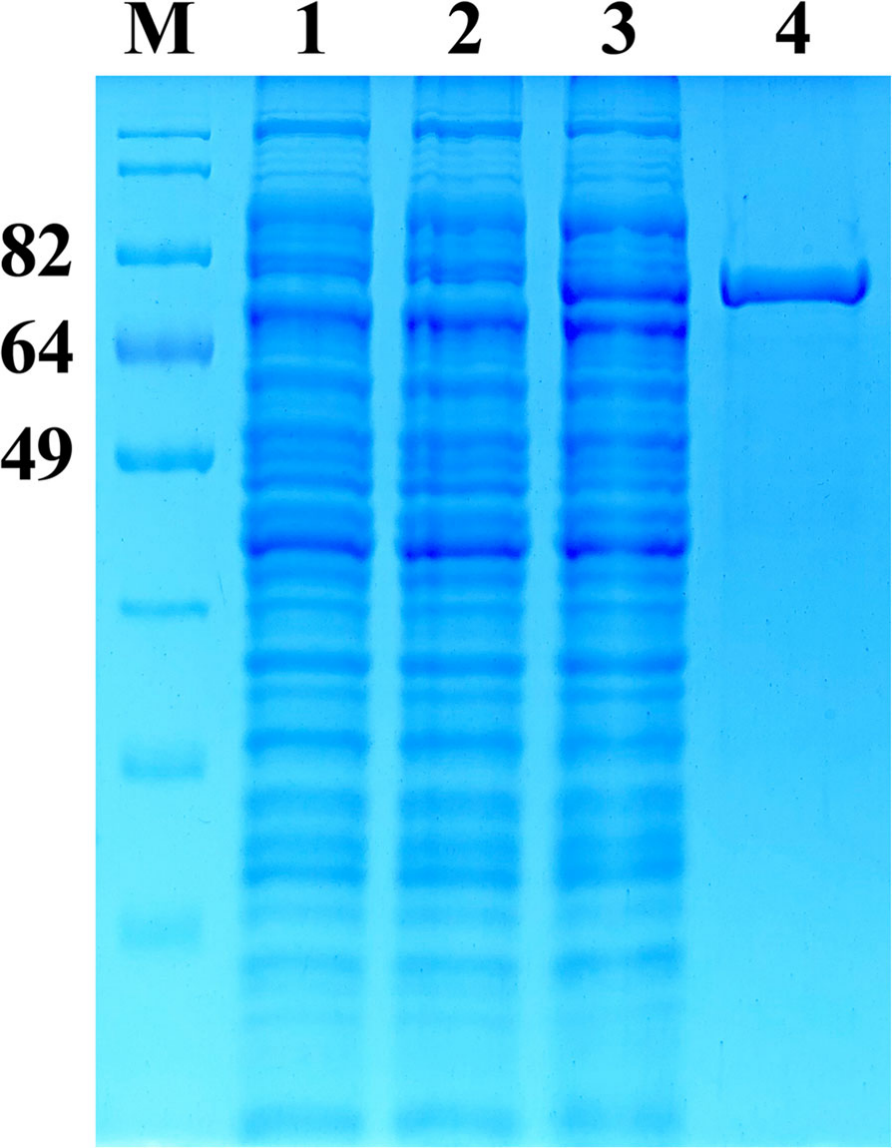
Purification of the rRHT-D1A protein of wheat variety Saratovskaya 29. Lane M: standard protein molecular weight markers; lane 1: a protein lysate from untransformed *E. coli*; lane 2: an *E. coli* protein lysate prior to induction; lane 3: the lysate of the same cells with IPTG induction; lane 4: the purified rRHT-D1A protein

The pET-28c-*Rht-D1a* plasmid was transformed into *E. coli* cells followed by cultivation in a standard manner by the method described in the “Methods” section. Protein expression was monitored by SDS-PAGE analysis. The total amount of protein produced by *E. coli* BL21 (DE3) and Rosetta-gami 2(DE3) was lower, and the protein of interest remained mostly in the insoluble fraction. Therefore, these competent cells were not used in further experiments. The most soluble protein was achieved by expression in *E. coli* Arctic Express (DE3) competent cells as a host. From the protein solubility point of view, 16 h of postinduction incubation at 13 °C was found to be optimal for producing the most soluble protein. In SDS-PAGE analysis, one major band with apparent molecular weight 70 kDa revealed the main difference between the bacterial cell lysates before and after the induction with IPTG. This band clearly emerged after the induction in all the chosen clones carrying the rRHT-D1A gene (Fig. 2, lane 3). The RHT-D1A protein fused with 6xHis at the N terminus was purified from the bacterial cell lysate by affinity chromatography on the HisTrap HP 1 ml column (GE Healthcare). The 6xHis-tagged RHT-D1A fusion protein appeared as a single band (after purification by affinity chromatography and SDS-PAGE) with an approximate molecular weight of 70 kDa (Fig. 2, lane 4). The yield of the recombinant 6xHis-RHT-D1A protein was 4 mg/L of bacterial culture.

### Production of the polyclonal antibody

**Fig. 3:**
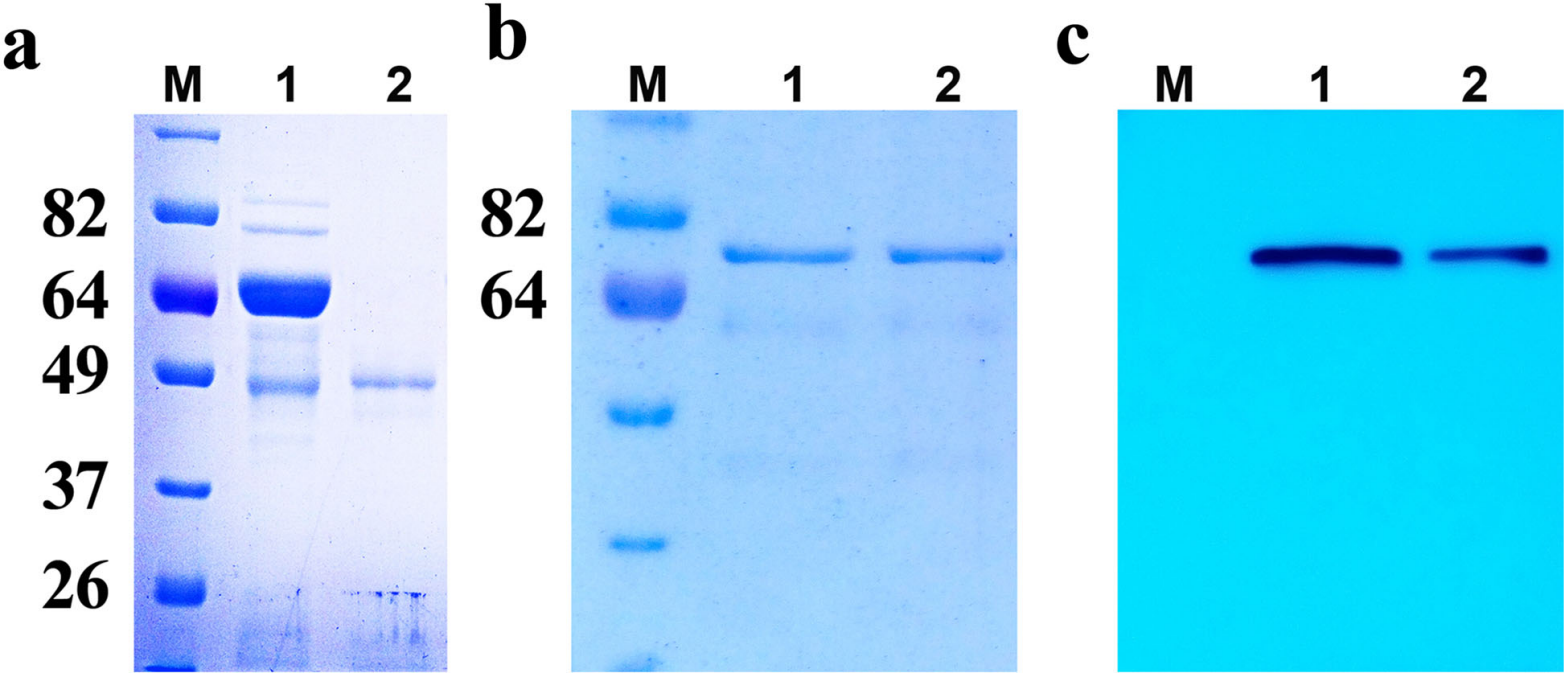
Purification of the rabbit anti-RHT-D1A polyclonal antibody and a western blot assay. **a** Purification of the rabbit anti-RHT-D1A polyclonal antibody. M: standard protein molecular weight markers; lane 1: immune serum (3 μg of protein); lane 2: the eluate from protein A–agarose (3 μg of protein). **b** SDS-PAGE of affinity-purified recombinant protein 6xHis-RHT-D1A. **c** The western blot assay. Lane 1: 3 μg of protein; lane 2: 1 μg of protein

**Fig. 4:**
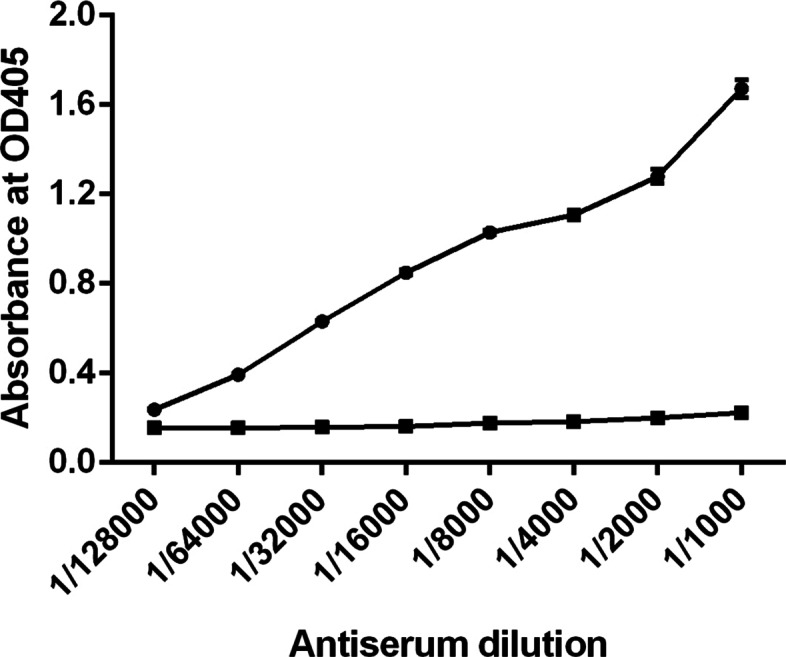
The titer of antiserum according to the ELISA. The purified antibody was subjected to serial dilution (from 1000- to 128000-fold) and reacted with the purified rRHT-D1A protein. Preimmunization rabbit serum served as a negative control. The antibody titer is defined as the highest dilution of serum at which the *A*_405_ ratio (*A*_405_ of postimmunization serum/*A*_405_ of preimmunization serum) is greater than 2:1. Data are presented as the mean + standard deviation

The purified 6xHis-RHT-D1A protein was employed as an antigen to generate a polyclonal antibody against wheat RHT-D1A. After the rabbits were immunized four times with the purified 6xHis-RHT-D1A protein, a polyclonal antibody against rRHT-D1A was first precipitated with different concentrations of ammonium sulfate and was further purified on the protein A–agarose column, following the manufacturer’s instructions. The purified antibody showed high purity and consisted of two bands according to electrophoresis: one was the heavy chain (~ 50 kDa), and the other was the light chain (~ 25 kDa), as assessed by SDS-PAGE in a 10% gel (Fig. 3a). The titers of the obtained RHT-D1A antisera were tested by an indirect ELISA. The ELISA results indicated that the titer of the purified polyclonal anti-RHT-D1 antibody is 1:64,000, suggesting that the purified polyclonal antibody has good sensitivity to rRHT-D1A (Fig. 4). The antibody titer is defined as the highest dilution of antiserum at which the ratio of *A*_405_ (*A*_405_ of postimmunization serum/*A*_405_ of preimmunization serum) is > 2:1.

### Evaluation of the anti-RHT-D1A polyclonal antibody by immunoblotting and IP

Western blot analysis was conducted to validate the reactivity and specificity of the purified polyclonal antibody by means of various amounts of purified rRHT-D1A protein (1 and 3 μg). The purified antisera reacted at different dilutions (1:1000 to 1:30,000) with an equal amount of the corresponding recombinant proteins. No positive signal was detectable in the preimmunization rabbit serum, which acted as a negative control. Western blotting with the anti-RHT-D1A antibody revealed an intense band at the expected position (Fig. 3c).

**Fig. 5:**
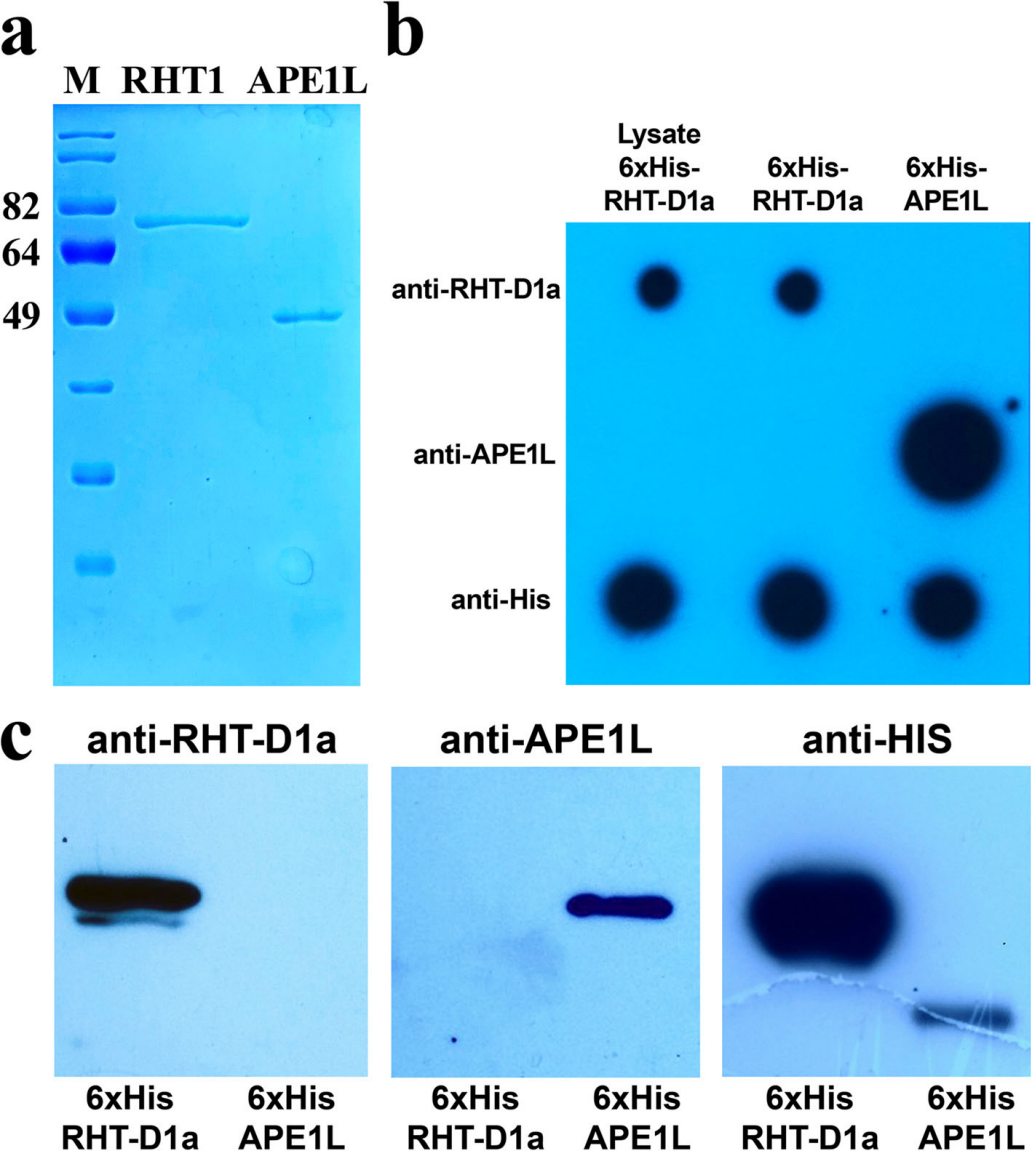
SDS-PAGE and immunoblot analysis of the polyclonal anti-RHT-D1A antibody by means of native and denatured rRHT-D1A proteins. **a** Five hundred nanograms of each protein was subjected to SDS-PAGE in a 10% gel and visualized by Coomassie brilliant blue staining. M: standard protein molecular weight markers. **b** Dot blot analysis of the anti-6xHis-RHT-D1A antibody against the native 6xHis-RHT-D1A recombinant protein. The anti-RHT-D1A, anti-APE1L, and anti-His antibodies were evaluated by the dot blot assay of purified 6xHis-RHT-D1A, a lysate of *E. coli* cells expressing 6xHis-RHT-D1A, and purified 6xHis-APE1L, which were spotted on PVDF membranes. **c** Western blot analysis of the polyclonal anti-RHT-D1A antibody against denatured rRHT-D1A proteins. The anti-RHT-D1A, anti-APE1L, and anti-His antibodies were evaluated by western blot analysis of 6xHis-RHT-D1A and 6xHis-APE1L. In all three western blots, 1 μg of purified 6xHis-RHT-D1A was loaded in the first lane and 0.5 μg of purified 6xHis-APE1L in the second lane

It is important to verify whether the antibody is specific to a protein’s native, nondenatured state or to the denatured protein because the denaturing treatment of protein samples prior to SDS-PAGE may alter the exposure and availability of the epitope, thus affecting antibody-binding affinity [20]. The capacity of the generated polyclonal anti-RHT-D1A antibody to identify nondenatured forms of RHT-D1A was tested by an immunodot assay involving recombinant 6xHis-RHT-D1A. The purified recombinant 6xHis-RHT-D1A protein, which was used for rabbit immunization, and an *E. coli* lysate containing the 6xHis-RHT-D1A protein were dotted on a PVDF membrane. A purified 6xHis-tagged recombinant APE1L protein [21], which was cloned and tagged in the same way as the RHT-D1A protein was, was dotted as a negative control. These control proteins were also utilized to test specificity and to rule out the possibility that the polyclonal antibody recognizes the 6xHis tag. The purified 6xHis-tagged rRHT-D1A and APE1L proteins were first analyzed by SDS-PAGE in gels stained with Coomassie brilliant blue (Fig. 5a) and then by dot and western blot hybridization (Fig. 5b, c).

The dot blots of the *E. coli* lysate containing 6xHis-RHT-D1A and purified recombinant 6xHis-RHT-D1A yielded strong signals with the polyclonal anti-RHT-D1A antibody. The dot blots of the control 6xHis-APE1L recombinant protein tested negative, whereas the anti-6xHis antibody recognized both proteins: 6xHis-RHT-D1A and 6xHis-APE1L. Next, we evaluated the ability of the polyclonal anti-RHT-D1A antibody to bind denatured RHT-D1A proteins by western blot analysis. The polyclonal anti-RHT-D1A antibody recognized denatured 6xHis-RHT-D1A but not the denatured recombinant 6xHis-APE1L protein (Fig. 5c).

Thus, the dot blot and western blot analyses indicated that the anti-RHT-D1A antibody recognizes both a nondenatured and denatured RHT-D1A protein. Hence, these results also rule out the possibility that the anti-RHT-D1A polyclonal antibody recognizes the 6xHis tag, which was present on the 6xHis-tagged RHT-D1A immunogen used to generate the polyclonal antibody.

The IP assay is an important tool for targeted protein purification, analysis of protein–protein interactions, and identification/analysis of protein complexes. A specific antibody that can selectively bind to the target epitope with high affinity is required for IP. For example, for some IP applications, the antibody should recognize the target protein in its native conformation; this property requires the accessibility of an epitope on the surface of the target protein [22].

**Fig. 6:**
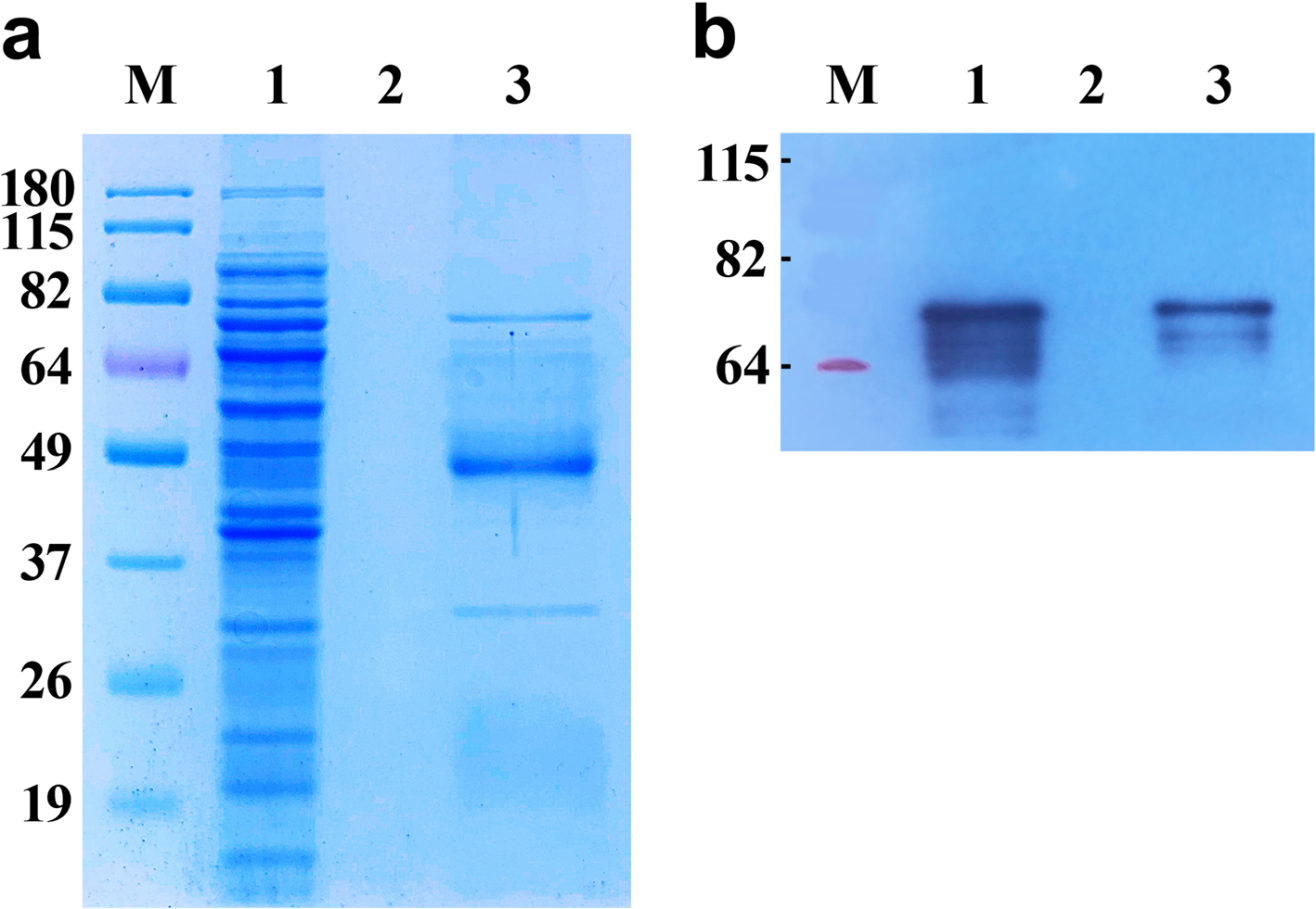
IP of the 6xHis-RHT-D1A recombinant protein by the polyclonal anti-RHT-D1A antibody. The immunoprecipitate was tested for 6xHis-RHT-D1A in a lysate of the *E. coli* strain expressing the recombinant protein. **a** Identification of 6xHis-RHT-D1A by SDS-PAGE in a 10% gel. The gel was stained with Coomassie brilliant blue. M: standard protein molecular weight markers; lane 1: the cell lysate; lane 2: the immunoprecipitate of a preparation not incubated with the antibody (control); and lane 3: the immunoprecipitate of the lysate treated with the polyclonal anti-RHT-D1A antibody. **b** Identification of 6xHis-RHT-D1A by western blot analysis with the polyclonal anti-RHT-D1A antibody. Lanes are the same as in Fig. 6**a**

The anti-RHT-D1A antibody was assessed in the IP of rRHT-D1A. The IP of RHT-D1A was performed in the cell lysate with protein G–agarose beads. As a control, the same IP procedure was performed with protein G–agarose beads lacking antibodies. The polyclonal anti-RHT-D1A antibody precipitated the 70-kDa rRHT-D1A protein. Proteins that were 25 and 50 kDa also stained in SDS-PAGE gels and represent antibody heavy and light chains (Fig. 6a). Western blot hybridization also revealed that the polyclonal anti-RHT-D1A antibody precipitated RHT-D1A (Fig. 6b). Control precipitate samples did not yield any significant bands either in SDS-PAGE with staining with Coomassie brilliant blue (Fig. 6a) or in western blots (Fig. 6b).

Thus, these data provided additional evidence that the obtained anti-RHT-D1A antibody can be used to detect the RHT-D1A protein in both undenatured and denatured states as validated by IP and dot and western blots.

### Detection of RHT-D1A in wheat seedlings using the polyclonal anti-RHT-D1A antibody

Our final goal was to obtain an antibody that could serve as a sensitive tool with which we can measure the endogenous level of an RHT-D1A protein in a tissue where this protein normally operates. Therefore, the final checkpoint for the newly raised polyclonal antibody was to determine whether this antibody specifically detects the endogenous RHT-D1A protein.

**Fig. 7:**
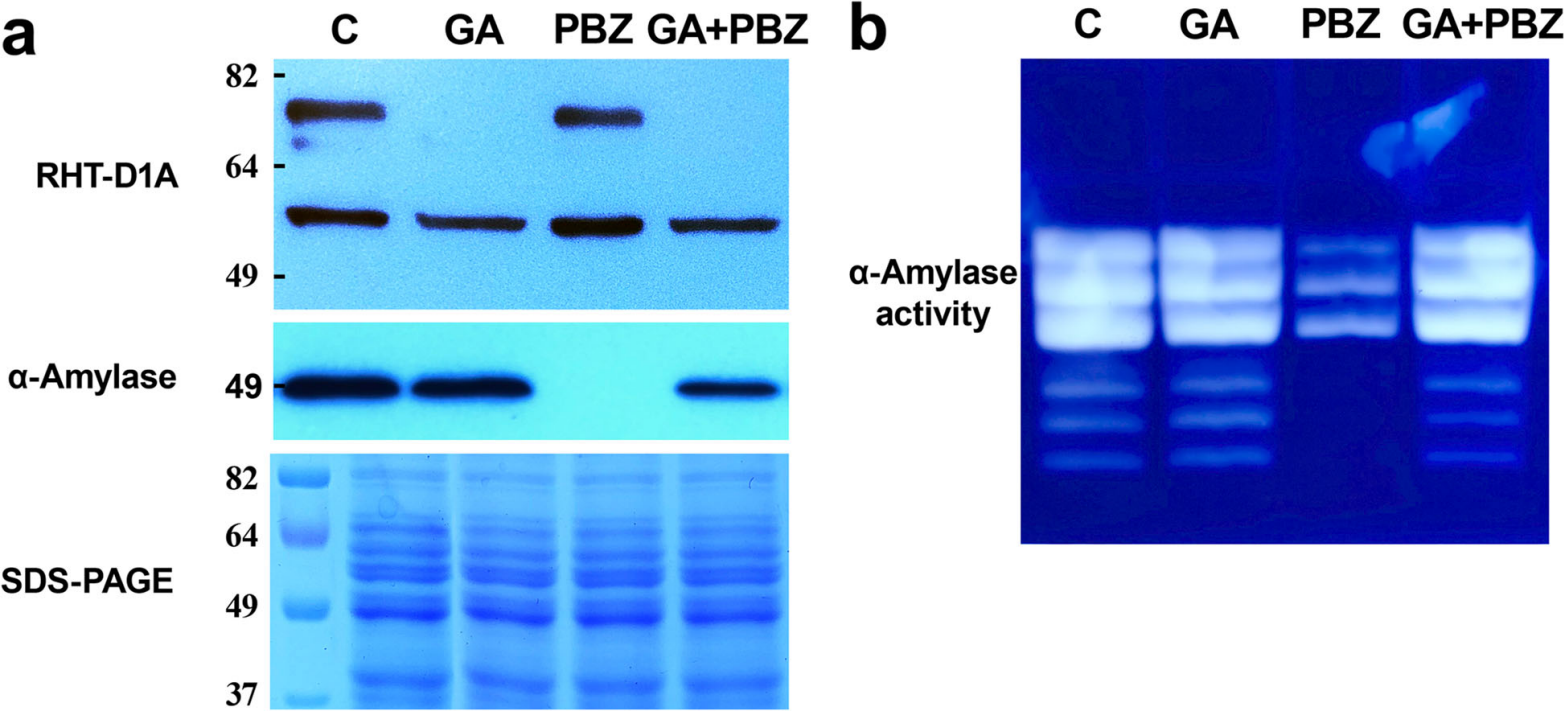
The effect of the GA biosynthesis inhibitor on the stability of RHT-D1A and on α-amylase production. **a** Western blotting of RHT-D1A and α-amylase. **b** Activity staining of α-amylases with native PAGE. Extracts were prepared from 4-day-old endosperm of wheat seedlings treated with 10 μM GA with or without 100 μM PBZ

Because DELLA protein degradation upon GA engagement by receptors called GID is the main regulatory mechanism in GA signaling, we analyzed the effect of a GA biosynthesis inhibitor, PBZ, on RHT-D1A stability. For this purpose, wheat seeds were germinated with water or 10 μM GA in the presence or absence of 100 μM PBZ. As shown in Fig. 7, inhibition of GA synthesis completely blocked α-amylase production and activity, indicating that the inhibitor successfully repressed the synthesis of gibberellins. Furthermore, the finding that PBZ blocked the α-amylase response in 4-day-old endosperm of wheat seedlings treated with water but did not inhibit the α-amylase production in the presence of GA meant that the observed effects of these inhibitors on GA responses are not attributable to nonspecific effects or the poisoning of cellular metabolism.

The anti-RHT-D1A antibody recognized 70- and 55-kDa proteins in the PBZ-treated seedlings. The 70-kDa protein is close to the predicted molecular mass of the endogenous RHT-D1A protein (65.3 kDa). We found that the 70-kDa protein band was not detectable in GA-treated wheat seedlings, confirming the high affinity of the polyclonal anti-RHT-D1A antibody. Most likely, the 55-kDa protein is unrelated to RHT-D1A because this protein was present in the GA-treated seedlings too. Consequently, our data clearly demonstrate that RHT-D1A disappears in response to exogenous and endogenous GA, as previously described for barley SLN1 [23] and rice SLR1 [24] proteins.

## Discussion

DELLA proteins play an essential part in the regulation of plant growth and adaptation to unfavorable environmental conditions [8]. In wheat, the majority of DELLA-regulated processes affect the seed yield and quality [25, 26]; accordingly, understanding the molecular basis of DELLA function holds great promise for improvement of the crop yield. To date, most studies in this field have dealt with a phenotypic effect of dwarf mutants of the *Rht* gene on yield-related traits, and researchers have not expressed the DELLA proteins of *Triticum* species. Furthermore, to further reveal the functions of the wheat RHT protein, it is necessary to have a highly sensitive and specific antibody against RHT. Accordingly, in this study, we prepared and characterized an anti-RHT-D1A polyclonal antibody.

In an initial experiment, with primer combinations specific for the tall and dwarfing alleles, we showed that the Saratovskaya 29 wheat variety does not carry mutant alleles *Rht-B1b* and *Rht-D1b*. Subsequently, we synthesized cDNA encoding the putative wheat RHT-D1 protein, and sequencing of the synthesized cDNA confirmed that the chosen wheat variety does not carry the mutant *Rht-D1* allele.

Previously, N-terminal domains of DELLA proteins from both *Arabidopsis* and *Malus domestica* have been isolated and expressed in *E. coli*. To ensure strong immune responses to small antigens such as the N-terminal domain of DELLA proteins, it is essential that these antigens be expressed as soluble proteins [27]. Of note, in that study, all the N-terminal domains of DELLA proteins were expressed at adequate levels of soluble proteins when MBP was employed as the protein fusion partner [27]. In our study, the most soluble full-length 6xHis-tagged rRHT-D1A was obtained by protein production in *E. coli* ArcticExpress (DE3) competent cells as a host. Soluble recombinant 6xHis-RHT-D1A was easily purified and was applied as a potent immunogen for the production of the anti-RHT-D1A polyclonal antibody.

The usual dose of a soluble protein administered with Freund’s adjuvant to rabbits is in the range of 50 to 1000 μg, and for mice, it is 10–200 μg; for goats or sheep, the typical dose is 250–5000 μg [28]. Nevertheless, for primary injection, most investigators use doses 100–200 μg [29, 30] or even less than 25–50 μg [31, 32]. It should be stressed that in some studies concerning the polyclonal antibody production in rabbits, for primary immunization, researchers have used doses of 400 to 500 μg [33, 34] even up to 1.0 mg [35, 36]. These observations point to the dependence of the antigen concentration required for primary immunization on immunogenic potency of the antigen. In our study, for the primary immunization, ~ 1 mg of the protein in Freund’s complete adjuvant was inoculated subcutaneously. The resultant polyclonal antibody was precipitated with 50% ammonium sulfate and further purified by affinity chromatography on protein A–agarose. Our indirect ELISA showed that the purified polyclonal antibody has good sensitivity to the rRHT-D1A protein (Fig. 4).

It is known that the specificity of an antibody is in part dependent on the type of an immunogen: a peptide or purified protein. Nonetheless, an antibody raised against a peptide may not work well when the respective protein in an immunoblot is in its native conformation. Such antibodies may not be useful for IP experiments but may bind the protein of interest after it is fully denatured. The opposite may also be true, especially if the immunogen was a purified protein, namely, an antibody works well for the protein in its native conformation but not when denatured [37].

Our anti-RHT-D1A polyclonal antibody successfully recognized the nondenatured protein in dot blot and IP analyses. Furthermore, the anti-RHT-D1A polyclonal antibody yielded strong signals by recognizing denatured RHT-D1A in western blotting. Here, this antibody was found to be rather specific to RHT-D1A because in dot and western blotting, the antibody did not react with the control recombinant TaAPE1L protein that was expressed and tagged in the same way as the RHT-D1A protein was [21] (Fig. 2).

In 4-day-old endosperm of wheat seedling extracts, the endogenous RHT-D1A protein was detected as a single band of the expected molecular mass, but the anti-RHT-D1A polyclonal antibody also reacted with another protein with molecular mass 55 kDa (Fig. 7). The 70-kDa protein degraded after treatment of plants with GA, as previously reported for barley (SLN1) and rice (SLR1) DELLA proteins [23, 24]. Our antibody recognized epitope(s) on the 55-kDa protein in all treatment groups, and the protein in question was not degraded in the presence of GA. Of note, in rice, two sequences have been identified that are homologous to SLR1: SLR1-like 1 and SLR1-like 2 (SLRL1 and SLRL-2). SLRL1 and SLRL2 contain regions with high similarity to the C-terminal conserved GRAS domains of SLR1 but lack the N-terminal conserved region of the DELLA proteins. DELLA proteins are members of the GRAS family of transcriptional regulators [38] containing two distinct domains: an N-terminal regulatory domain and a C-terminal functional GRAS domain. The N-terminal domain is required for binding the GID1–GA receptor complex, a process that ultimately triggers DELLA degradation and promotes GA-responsive growth. Furthermore, in contrast to DELLA proteins, SLRL1 is not degraded upon GA treatment, and SLRL1 overexpression is not responsive to GA [39]. Moreover, no sequences have been found in the *Arabidopsis* genome that are homologous to SLRL1 and SLRL2. Given that maize contains a sequence highly similar to SLRLs, it has been suggested that SLRL-type GRAS proteins exist in monocots but not dicots [39]. Although we do not know the identity of the 55-kDa protein, we can speculate that it may be a wheat protein homologous to SLRL1 and SLRL2. Further studies are needed to identify the 55-kDa protein. On the other hand, we cannot rule out the presence in RHT-D1A of a specific proteolytic cleavage site that can generate a truncated form of RHT-D1A. The biological activity of many proteins, including transcription factors, is regulated by post-translational modifications involving a controlled proteolytic activity. Proteolysis can remove segments of a protein to enable or prevent its biological function and in addition may cause changes of subcellular localization by removing localization sequences [40].

Although bacterially expressed rRHT-D1A should not have post-translational modifications and an optimal folding environment present in plants, this situation did not decrease the immunogenicity of this recombinant protein in rabbits. Our results indicate that the use of the rRHT-D1A antigen generated in the bacterial expression system did not lead to substantial alterations in the antigen–antibody recognition reaction for RHT-D1A. The purified polyclonal antibody raised against the RHT-D1A protein is sensitive and useful for research on DELLA-regulatory mechanisms in wheat.

## Conclusion

The PCR analysis with allele-specific primers and sequencing of the synthesized cDNA of RHT-D1A confirmed that the Saratovskaya 29 wheat variety does not carry mutant alleles *Rht-B1b* and *Rht-D1b*. The synthesized cDNA of RHT-D1A was successfully expressed in an *E. coli* system, and the affinity-purified wheat protein was utilized as an immunogen to prepare the antibody against the RHT-D1A protein. The polyclonal antibody has high sensitivity and satisfactory specificity for the detection of RHT-D1A by dot blot hybridization, western blotting, and IP; therefore, it should open new opportunities for further studies on the molecular mechanism of action of DELLA in this highly important group of plants.

## Data Availability

Not applicable

## Abbreviations

*A*_405_: Absorbance at 405 nm
Ape1L: Apurinic/apyrimidinic endonuclease
cDNA: Complementary deoxyribonucleic acid
DNase: Deoxyribonuclease
DTT: Dithiothreitol
EDTA: Ethylenediaminetetraacetic acid
*E. coli*: *Escherichia coli*
GA: Gibberellic acid
GST: Glutathione S-transferase
IgG: Immunoglobulin G
IP: Immunoprecipitation
IPTG: Isopropyl β-d-1-thiogalactopyranoside
MBP: Maltose-binding protein
mRNA: Messenger ribonucleic acid
OD_600_: Optical density at 600 nm
PBZ: Paclobutrazol
PBST: Phosphate-buffered saline with Tween 20
PCR: Polymerase chain reaction
PVDF: Polyvinyl difluoride
rRHT-D1A: 6xHis-tagged recombinant RHT-D1A
Rht: Reduced height
RIPA: Radioimmunoprecipitation assay buffer
RNase: Ribonuclease
SDS-PAGE: Sodium dodecyl sulfate and polyacrylamide gel
TBS: Tris-buffered saline
TBST: Tris-buffered saline with 0.005% of Tween 20
UV: Ultraviolet
